# Variable expressivity familial cherubism: woman transmitting cherubism without suffering the disease

**DOI:** 10.1186/1746-160X-9-33

**Published:** 2013-11-05

**Authors:** Mario Pérez-Sayáns, Francisco Barros-Angueira, José M Suárez-Peñaranda, Abel García-García

**Affiliations:** 1 Oral Medicine, Oral Surgery and Implantology Unit, Faculty of Medicine and Dentistry, Institute of Sanitary Research of Santiago, (IDIS), Entrerrios s/n, Santiago de Compostela, CP 15782, Spain; 2 Molecular Medicine Unit-Galician Public Fundation for Genomic Medicine, University Hospital and School of Medicine of Santiago de Compostela, Santiago de Compostela, Spain; 3 Department of Pathology and Forensic Sciences, University Hospital and School of Medicine of Santiago de Compostela, Santiago de Compostela, Spain

**Keywords:** Cherubism, SH3BP2, Expressivity

## Abstract

Cherubism is classified within the group of benign osteo-fibrous lesions. Aside from facial deformities, it may account for major complications. It has been observed that the disease is caused by a mutation in the gene SH3BP2 (SH3-domain binding protein 2), which is located at chromosome 4pl6.3. Here we present two cases of familial cherubism, uncle and nephew, with variable clinical involvement (“Expressivity”), and one case of a woman (sister and mother, respectively), who transmitted cherubism without suffering the disease. In this article we have shown that, in familial cherubism cases, the mutation is inherited through an autosomal dominant transmission. Mutations affecting gene SH3BP2 cause variable clinical involvement (variable expressivity), involvement can be moderate, severe or may result merely in asymptomatic carriers. Since the possibility of transmission reaches 50% of chances, we believe that it is important to develop genetic counseling for both patients and carriers, in order to prevent or minimize new affected offspring.

## Introduction

Cherubism, listed by OMIM (Online Mendelian Inheritance in Man) as OMIM 118400, is a rare genetic disease of dominant autosomal inheritance with variable penetrance and expressivity, characterized by an abnormal growth of the bones of the face, mainly the jaw [1]. Prevalence is unknown, but is probably less than 1 in 10,000 [2]. In general, it appears around age 4 with painless bilateral swelling of the jaws (which create a cherubim-like look) and stops progression in the late teens, with residual manifestations persisting in adult life [3]. The term cherubism was suggested to describe the appearance of the patients due to their resemblance to a Renaissance cherub [4].

Cherubism is classified within the group of benign osteo-fibrous lesions, differentiating from osteo-cementum lesions and fibrous dysplasia because of its unique clinical and radiological features [1]. Besides facial deformities, there may be complications, including vision loss due to optic neuropathy, obstruction of upper airways, sleep apnea, language disorders and difficulty chewing due to alterations in the appearance and development of teeth [5]. Teething is also abnormal and dental agenesis, lack of eruption, displacement, root resorption and malocclusions are common problems. X-ray imaging contributes to the diagnosis showing multiocular radiolucencies that are well defined and, with age, thick sclerotic lines, usually symmetrical [6]. The definitive diagnosis is made histologically, with the appearance of randomly distributed multinucleated giant cells and vascular spaces within the stroma of the fibrous connective tissue. These multinucleated giant cells are positive for osteoclast-specific markers [7].

It has been observed that the disease is due to a mutation in gene SH3BP2, which is located at chromosome 4pl6.3, which encodes a protein formed by 561 amino acids [8]. In patients with cherubism, mutations have only been described in exon 9 of gene SH3PB2. To date, mutations in the other exons of this gen have not been described. It has also been related to a range of other diseases such as Noonan and Noonan-like syndromes (caused by mutations in gene PTPN11), and Ramon’s syndrome, which is related to gingival fibromatosis and type I neurofibromatosis [9].

**Figure 1:**
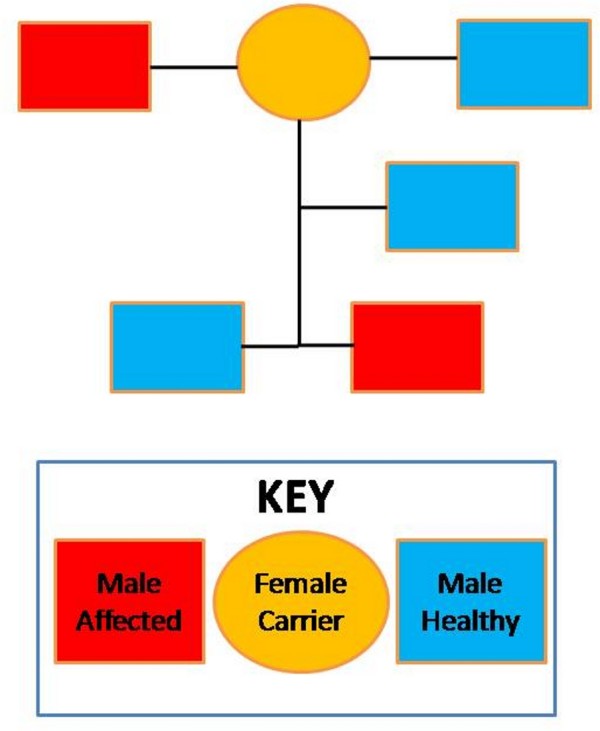
**Family involvement scheme of cherubism disease.** Arrow: polypoid formation in the right maxillary sinus. Asterisks: multiple epidermal cysts.

Here we present two cases of familial cherubism, uncle and nephew (hereinafter, Patient 1 and 2, respectively) (Figure 1), with variable clinical involvement (“Expressivity”), together with the radiological description and the molecular confirmation of the case through the detection of a mutation in gene SH3BP2 (SH3-domain binding protein 2). After PCR, the entire coding region including intron-exon boundaries of the exon 9 of the SH3BP2 gene was analyzed by bidirectional sequencing (Big Dye Terminator v3.1, LifeTechnologies) using an ABI3730XL Analyzer (Applied biosystems, Foster City, CA, USA).

## Consent

Written informed consent was obtained from the patients for publication of this case report and any accompanying images. A copy of the written consent is available for review by the Editor-in-Chief of this journal. The present study was approved by the CHUS research ethics committee.

## Results

**Figure 2:**
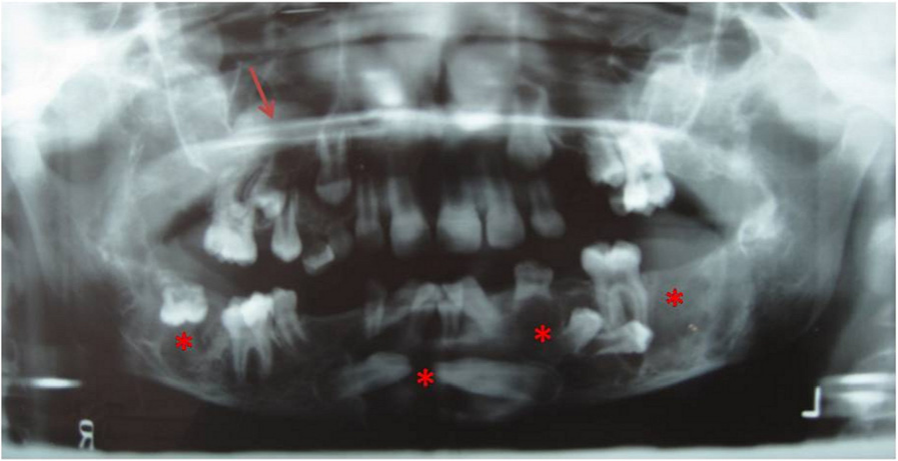
Panoramic radiography of the uncle affected.

Our patients are a man (Patient 1), the first familial cherubism case, which was diagnosed in the year 1985 at the age of 9 years; and his nephew (Patient 2), who was diagnosed with cherubism in 2013 at the age of 7 years. Patient 1, who is currently 37 years old, visited the Maxillofacial Surgery Unit due to severe tooth malpositioning. After radiological examination using panoramic X-rays, we confirmed the presence of multiple epidermal cysts with numerous malpositioned teeth, and a polypoid formation in the right maxillary sinus (Figure 2). We performed an extraction of all lower teeth except for piece 36 and the deep inclusions of premolars (34 and 35) and we performed curettage of the bone lesions. We collected biopsies from soft and hard tissue and teeth. The anatomical and pathological report confirmed fibroblastic lesions with a prominent vascular pattern, dotted with abundant multinucleated giant cells and newly formed bone in the peripheral areas, which supports cherubism after discarding the brown tumor of hyperparathyroidism. Patient 1 also had orbital involvement, and microstrabismus and neuropathic vision loss. The orbital involvement required inferior orbitotomy with resection and orbital floor removal and placement of a Medpor Orbital Reconstruction Implants prosthesis (Stryker, Kalamazoo, USA) to reinforce the orbital floor. According to the Raposo-Amaral classification [10], it would be grade VI, due to the rare involvement of the jaw, maxillary sinus and orbit.

**Figure 3:**
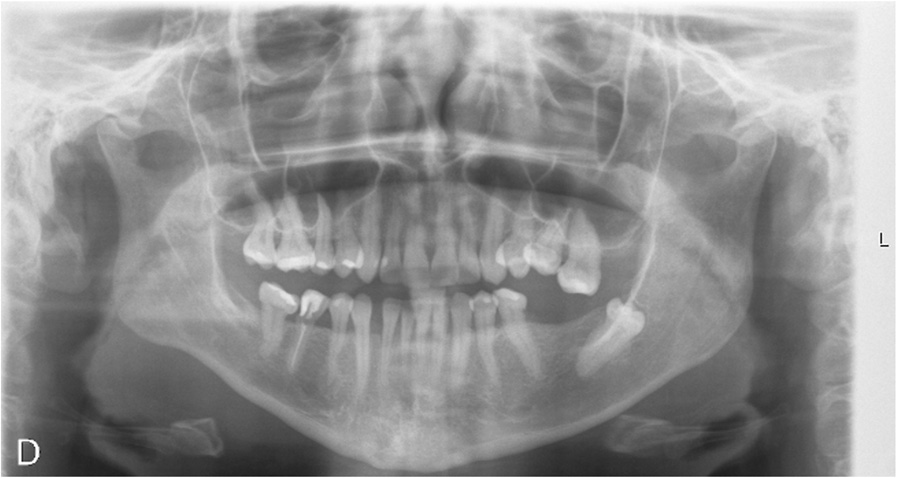
Panoramic radiography of the sister, non-affected.

**Figure 4:**
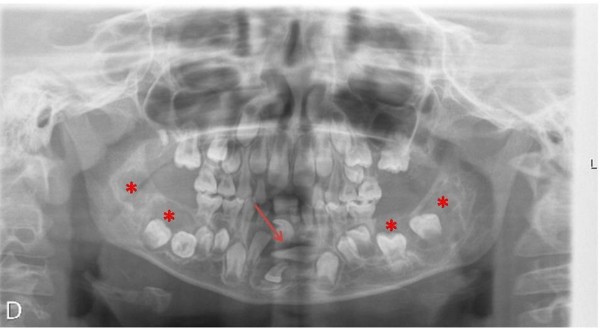
**Panoramic radiography of the nephew, affected.** Arrow: follicular cyst in piece 43. Asterisks: odontogenic cysts in posterior mandibular regions and right mandibular area.

Patient 1 has two siblings, a man and a woman, neither of them is affected by the disease. The sister (Figure 3) has two children. Her firstborn (Patient 2) is 8 years old. He visited the Maxillofacial Surgery Unit due to the delay of the eruption of permanent incisors without suspecting any relation to his uncle’s pathology. Upon radiological examination, we observed the lack of eruption of permanent upper teeth, germination of lower central incisors, follicular cyst inclusion in piece 43, horizontal ectopic inclusion of tooth 32, supernumerary jaw, and odontogenic cysts in posterior mandibular regions and right mandibular area (Figure 4). We immediately suspected familial cherubism. Furthermore, Patient 2 referred nonspecific headaches that disappeared with nonsteroidal anti-inflammatory drugs, so we evaluated a possible ocular involvement, which has not been confirmed yet. According to the Raposo-Amaral classification, it would be grade III, class 5. Currently he is eating enteral nutrition, O-LAC (Mead Johnson).

In these two clinical cases, we decided to conduct a confirmatory molecular study of the uncle (Patient 1), mother, and her firstborn son (Patient 2) and develop a predictive study of Patient 2′s younger brother, who is now 5 years old and clinically asymptomatic. EDTA blood samples were collected for sequencing analysis in all patients. The analysis consisted of the capillary sequencing of the amplified exon 9 of gene SH3BP2.

For Patient 1, with extreme phenotype, we observed a genotype: SH3BP2 R415Q/normal (in HGVS nomenclature: c. [1244G > A], [=]), showing that the mutation of R415Q causes a change in the Arg amino acid by Gln at position 415 of the protein and is described as pathologic with reduced penetration and variable expression. This result confirms the diagnosis of cherubism in which the chances of transmitting the mutation amount to 50%. Meanwhile, in the mother, with healthy phenotype, we also observed a SH3BP2 R415Q/normal genotype with the same likelihood of transmission. The eldest son, the sibling affected by an extreme phenotype, showed the same genotype SH3BP2 R415Q/normal. However, the youngest son, who is phenotypically asymptomatic, presented a SH3BP2 normal/normal genotype and does not carry the mutation. Therefore he is not at risk of developing the disease or transmitting it.

## Discussion

Cherubism is an autosomal dominant disease, with variable expressivity (different from the classical concept of “penetrance”) [11]. By sex, expressivity amounts to nearly 100% in men, and shows reduced expression in women ranging from 50 to 75%, although sporadic cases of cherubism have been reported [12,13]. This can be seen in the family that we describe, both men have clearly established cherubism, while the woman is completely asymptomatic.

Clinically there is a painless enlargement of the lower half of the face, which starts approximately at age 3 or 4 and continues through to adolescence. In most cases this growth stops at the end of adolescence, thus the typical appearance of cherubism disappears when these lesions regress around age 20 and residual lesions are not recognizable. Residual deformity of the jaw is uncommon. The rest of the skeleton is not affected, and markers of bone remodeling are within normal range [3,7]. Raposo-Amaral [10], presents a clinical classification of VI degrees of expression of the disease. We believe that this classification is useful when comparing the clinical situation of patients. However, we wish to present a modification of this classification by introducing degree 0, which would include individuals who inherit the mutation of SH3BP2 but do not present any type of expression of the disease (Table 1). Therefore, in regards of the members of the family presented here, and according to our classification, the mother would be grade 0, the uncle would be grade VI, whereas the nephew would have grade III.

**Table 1:** **Proposed clinical classification for cherubism, modified from Raposo-Amaral classification**[10]

**Grade**	**Definition**
**0**	Existence of the mutation without expression of the disease.
**I**	Lesion of the mandible without signs of root resorption
**II**	Lesions involving the mandible and maxilla without signs of root resorption
**III**	Aggressive lesion of the mandible with signs of root resorption
**IV**	Lesions involving the mandible and maxilla with signs of Root resorption
**V**	The rare, massively growing, aggressive, and extensively deforming juvenile lesions involving the maxilla and mandible
**VI**	The rare, massively growing, aggressive, and extensively deforming juvenile lesions involving the maxilla, mandible, and orbits

The differential diagnosis must be made with fibrous dysplasia, odontogenic cyst, juvenile ossifying fibroma, giant cell granuloma, fibrous osteoma, osteosarcomae hyperparathyroidism. The histopathology is characterized by a large number of multinucleated giant cells, scattered throughout the fibro-osseous connective tissue, with the presence of spindle cells and osteoid trabeculae [14].

We must clarify a persistent error in many of the papers dealing with cherubism, which affirm that the lesions that appear in the jaws are cysts. This is absolutely false, since a cyst is a pathological cavity (often filled with liquid) that is surrounded by epithelium [15]. In cherubism, the lesions that insufflate the jaws are essentially tissue masses similar to giant cell granulomas. We believe that this error derives from the first description of the disease, which was coined by Jones: “Familial multilocular cystic disease of the jaws” [4].

Ueki et al. [8] identified mutations in gene SH3BP2, which caused cherubism and established the genetic basis for classifying the disease as a separate entity of fibrous dysplasia of the jaws. Gene SH3BP2 is located at 4p16.3 and transcribes a protein of 561 amino acids that binds to the SH3 domains of certain transducer proteins, having proven its expression in multi-nucleated cells and the stromal cells of fibrous tissue of cystic lesions of affected patients [16]. Although this mutation persists throughout the life of the patient, the expression of the related genes changes with age (unknown mechanism) and the existing lesions tend to auto-resolve in adulthood [2,6].

There have been reports of cherubism cases associated with Noonan syndrome without showing mutations at gene SH3BP2, which is considered as an expression of the disease rather than two related entities [9]. The association between Ramon’s syndrome [17] and the Jaffe-Campanacci [18] and the fragile X syndrome [19] has also been described. The most common genetic abnormality is the R415Q mutation that causes a change in amino acid Arg by Gln at position 415 of the protein [8]. Other mutations have been described for the same gene, with varying degrees of clinical involvement. Li et al. [20] describe the change of the base of A1517G that causes a substitution of the D419G amino acid. Carvalho et al. [21], describe a mutation corresponding to the deletion of cytosine (408 of C), located in the PH domain in SH3BP2. Lietman et al. [22] describe a missense mutation (aspartic acid to asparagine, p.D419N (g.1371G > A, c.1255G > A) in exon 9 of SH3BP2. Other mutations described are: p.D420E (c.1259G > A) [23], and p.P418R (c.1253C > G) [8].

Other hypotheses have been described in the pathogenesis of cherubism. Thus, Hyckel et al. consider that cherubism is a disorder induced by altered signals in the transduction of the parathyroid hormone due to the influence of protein SH3BP2 in the regulation of the union of the receptor of the parathyroid-hormone and Parathyroid hormone-related protein (PTHrP) [24].

The main complications described in the disease are optic neuropathy caused by orbital involvement, sleep, speech and chewing disorders and airway obstruction [5,7]. In the cases presented, one of the patients had orbital and ophthalmic involvement with no signs of optic neuritis, while the other patient has not presented any other problem aside from the dentoskeletal deformity of the jaws and the resulting difficulty chewing.

To treat cherubism, in Patient 1′s case, we have resorted to surgical decompression of the orbit and a reconstructive surgery of the jaws [3], but sometimes one can opt for watchfully waiting for the involution of the lesions that is expected by the end of adolescence. In the family that we studied, we were able to confirm the presence of the same mutation in the heterozygous genotype, resulting in a dominant inheritance and not a *denovo* mutation.

## Conclusions

In this article we have shown that, in familial cherubism cases, the mutation is inherited through an autosomal dominant transmission. Mutations affecting gene SH3BP2 cause variable clinical involvement (variable expressivity), involvement can be moderate, severe or may result merely in asymptomatic carriers. Since the possibility of transmission reaches 50% of chances, we believe that it is important to develop genetic counseling for both patients and carriers, in order to prevent or minimize new affected offspring.

## Competing interests

The authors declare that they have no competing interests.

## Authors’ contributions

MPS is responsible for the design and writing of the study. FBA has collaborated in the genomic analysis of the patients. JMSP specialist pathology has been responsible of the analysis of the specimens and immunohistochemical studies for the diagnosis. AGG is the group leader and is responsible for the idea of the study. All authors read and approved the final manuscript.
